# [Corrigendum] Predictive DNA damage signaling for low-dose ionizing radiation

**DOI:** 10.3892/ijmm.2025.5553

**Published:** 2025-05-21

**Authors:** Jeong-In Park, Seung-Youn Jung, Kyung-Hee Song, Dong-Hyeon Lee, Jiyeon Ahn, Sang-Gu Hwang, In-Su Jung, Dae-Seog Lim, Jie-Young Song

Int J Mol Med 53: 56, 2024; DOI: 10.3892/ijmm.2024.5380

Following the publication of the above article, the authors drew to the Editor's attention that two pairs of the data panels shown for the flow cytometric experiments in Fig. 3B on p. 7 had inadvertently been duplicated in the figure, such that these data were derived from the same original source, even though they were intended to have shown the results from different experiments.

The authors were able to consult their original data, however, and the revised version of Fig. 3, now containing the correctly assembled flow cytometric plots in Fig. 3B, is shown on the next page. Note that the errors made in assembling these figures did not affect the overall conclusions reported in the paper. All the authors agree with the publication of this corrigendum, and are grateful to the Editor of *International Journal of Molecular Medicine* for granting them the opportunity to publish this. Furthermore, they apologize to the readership for any inconvenience caused.

## Figures and Tables

**Figure 3:**
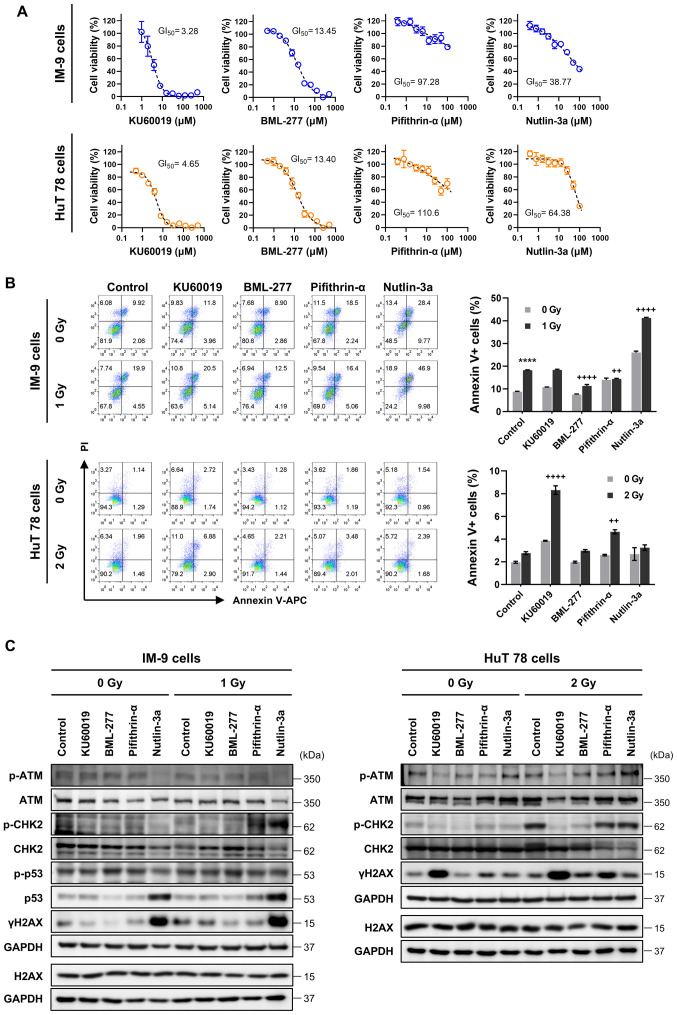
Protective effects of DNA damage repair modulators against IR-induced cell death. (A) Cells were serially diluted with the indicated compounds and treated for 24 h, and cell viability was then measured using CCK-8 assay. Data shown are the means ± SEM of three independent experiments. (B) IM-9 and HuT78 cells were treated with or without KU60019 (ATM inhibitor, 2.5 *µ*M), BML-277 (CHK2 inhibitor, 2.5 *µ*M), pifithrin-α (p53 inhibitor, 5 *µ*M) and nutlin-3a (p53 activator, 10 *µ*M) 24 h prior to irradiation. Cells were analyzed for Annexin V binding and for PI uptake using flow cytometry.

